# Efficacy and Cost-Effectiveness of Vonoprazan–Amoxicillin Dual Therapy Versus Esomeprazole–Bismuth Quadruple Therapy for *Helicobacter pylori*: A Propensity Score-Matched Study

**DOI:** 10.3390/microorganisms14051006

**Published:** 2026-04-29

**Authors:** Lihua Guo, Jiaxin Ge, Min Miao, Guimei Hu, Jinfeng Wen, Kefeng Hu, Guoliang Ye

**Affiliations:** Department of Gastroenterology, The First Affiliated Hospital of Ningbo University, Ningbo 315020, China; lihua2005wind@163.com (L.G.); fygejiaxin@nbu.edu.cn (J.G.); miaomin12@sina.com (M.M.); hgm930@139.com (G.H.); wenjjff@sina.com (J.W.); nbhkf@sina.cn (K.H.)

**Keywords:** *Helicobacter pylori*, dual therapy, bismuth-containing quadruple therapy, eradication rate, cost-effectiveness, probabilistic sensitivity analysis

## Abstract

Vonoprazan–amoxicillin (VA) dual therapy offers a simplified alternative; however, its efficacy and cost-effectiveness relative to bismuth-containing quadruple therapy (BQT) in treatment-naïve patients remain unclear. This study aimed to compare the eradication rate and cost-effectiveness between the two groups using a propensity score-matched analysis. This retrospective, single-center study used outpatient electronic medical records from January 2024 to November 2025. After propensity score matching (1:1), 212 patients received vonoprazan–amoxicillin dual therapy (VA), and 212 matched patients received esomeprazole–bismuth quadruple therapy (EBAC) for two weeks. Eradication rates and cost-effectiveness were compared. According to the intention-to-treat (ITT) analysis, eradication rates were 90.56% (95% CI 85.88–93.81%) in the VA group and 90.09% (95% CI 85.33–93.43%) in the EBAC group (*p* = 0.869). The per-protocol (PP) analysis showed eradication rates of 93.03% (95% CI 88.64–95.80%) and 91.58% (95% CI 86.93–94.68%), respectively (*p* = 0.585). Multivariate logistic regression identified non-compliance as a significant risk factor for eradication failure (OR = 5.346, 95% CI 1.847–15.473, *p* = 0.002). Probabilistic sensitivity analysis showed that EBAC was more cost-effective than VA in stage 1. However, the eradication rate of EBAC dramatically declined, and VA became the more cost-effective option at higher WTP thresholds in stage 2. In conclusion, the 14-day VA dual therapy is as effective as BQT therapy but is safer and easier to use, offering a more streamlined eradication option. Nonetheless, EBAC remains a cost-effective option.

## 1. Introduction

*Helicobacter pylori* (*H. pylori*), as a Gram-negative bacterium, is capable of surviving in the gastric acidic environment and colonizing the stomach [1]. As a pathogenic bacterium, *H. pylori* is closely associated with chronic gastritis and peptic ulcer disease [2]. Long-term infection significantly increases the risk of gastric mucosal atrophy, intestinal metaplasia, and even carcinogenesis [3].

In China, the estimated infection rate was 40.66% according to a large-scale epidemiological study, with even higher prevalence observed in certain regions [4]. Early recognition and management of *H. pylori* infection offer sustained benefits, including curing most cases of peptic ulcer disease and preventing its recurrence [5]. It greatly lowers the risk of gastric cancer. For those with pre-existing precancerous lesions, it can also slow disease progression and reduce the incidence of cancer [6,7].

Bismuth-containing quadruple therapy (BQT) is strongly recommended as the first-line approach for *H. pylori* eradication in China [8]. This strategy aligns with international guidelines for regions with high dual resistance [5]. Through the combined action of antibiotics, proton pump inhibitors (PPIs), and bismuth, BQT significantly increases eradication rates, reduces the risk of antibiotic resistance, and improves the gastric mucosal environment [5,9,10]. However, quadruple therapy often results in lower patient adherence, interruptions in treatment, and ultimately failure due to its complexity, which includes multiple medications, a complicated dosing schedule, and side effects [2,11].

Vonoprazan, as a novel potassium-competitive acid blocker (P-CAB), reversibly inhibits gastric H^+^/K^+^-ATPase. Compared with conventional proton pump inhibitors (PPIs) such as esomeprazole, it offers a faster onset, longer-lasting acid suppression, easier dosing, and more effective *H. pylori* eradication, supported by multiple clinical trials [12,13]. High-dose dual therapy (HDDT) has emerged as a promising and effective option for eradicating *H. pylori*, with success rates comparable to those of BQT [14,15,16]. The Maastricht VI/Florence Consensus Report (2022) concludes that P-CAB-based regimens are non-inferior or superior to conventional PPI-based therapies for both first- and second-line treatments. Furthermore, they demonstrate greater effectiveness in patients with documented antimicrobial-resistant infections [17]. The 2024 Clinical Practice Update from the American Gastroenterological Association (AGA) indicates that vonoprazan–amoxicillin (VA) dual therapy can be used as a first-line treatment option for treatment-naïve patients [2]. This superior efficacy may be attributed to P-CABs’ powerful and long-lasting acid-suppressive properties, as previously indicated. Its pharmacological onset is rapid, occurring within one hour after a single dose, significantly faster than that of conventional PPIs [18]. Although its plasma half-life is approximately 7–9 h, the suppression of gastric acid secretion persists for over 24 h. The 24 h holding time ratio (HTR) for intragastric pH > 4 exceeds 90%, demonstrating superior acid control compared to traditional PPIs [19]. Effective *H. pylori* eradication depends heavily on maintaining a high intragastric pH, as *H. pylori* thrives at pH 6.0–8.0, where antibiotics such as amoxicillin and clarithromycin are most effective [13]. Unlike PPIs, vonoprazan is primarily metabolized by CYP3A4 and is less influenced by CYP2C19 genetic polymorphism [12].

The VA regimen usually combines twice-daily vonoprazan with high-dose amoxicillin given three times a day for 10 or 14 days [20]. This regimen establishes a nearly sterile, alkaline gastric environment through intensive acid suppression. At the same time, high-dose, multiple-daily amoxicillin maintains consistent bactericidal plasma levels, enabling the continuous eradication of *H. pylori* [21]. In some areas with low amoxicillin resistance, HDDT achieves eradication rates comparable to those of optimized BQT, with additional benefits including minimized drug interactions, fewer adverse effects, good compliance, and a reduced risk of antimicrobial resistance [5,22].

Therefore, this study aimed to compare *H. pylori* eradication rates and cost-effectiveness between VA dual therapy and esomeprazole–bismuth–amoxicillin–clarithromycin (EBAC) quadruple therapy.

## 2. Materials and Methods

### 2.1. Study Design and Populations

The data of outpatients with *H. pylori* infection who received either VA dual therapy or EBAC quadruple therapy were analyzed retrospectively. Information was collected from the outpatient clinical workstations of the First Affiliated Hospital of Ningbo University between January 2024 and November 2025. *H. pylori* infection was diagnosed with a ^13^C/^14^C-urea breath test (UBT) or histopathological examination of gastric biopsy specimens obtained during endoscopy.

Inclusion and exclusion criteria are listed in Table 1. This study received ethical approval from the Institutional Review Board of the First Affiliated Hospital of Ningbo University (Protocol No. 2026-011RS).

### 2.2. Eradication Regimens for H. pylori Infection

The VA dual regimen included vonoprazan 20 mg twice daily before meals and amoxicillin 1000 mg three times daily after meals for 2 weeks. The EBAC quadruple regimen consisted of esomeprazole 20 mg and colloidal bismuth pectin 300 mg twice daily before meals, plus amoxicillin 1000 mg and clarithromycin 500 mg twice daily after meals, also for 2 weeks. *H. pylori* eradication status was reassessed using a UBT at least 4 weeks after completing therapy.

### 2.3. Propensity Score Matching

To adjust for confounding and selection bias in this non-randomized study, we rigorously implemented PSM to equilibrate baseline demographic, clinical, and procedural characteristics between the VA and EBAC cohorts. Variables that might potentially influence *H. pylori* eradication rates, including age, sex, endoscopic findings, and comorbidities, were incorporated as covariates into a binary logistic regression model. Matching was conducted using a caliper width of 0.02, with a 1:1 nearest-neighbor algorithm applied.

### 2.4. Cost-Effectiveness Analysis

The total medication cost for the VA group remained stable throughout the entire observation period in the cost-effectiveness analysis. Vonoprazan was officially added to the national reimbursement drug list for *H. pylori* eradication starting 1 January 2025, leading to a significant increase in the use of the VA regimen from that date. Conversely, a substantial decrease in drug costs was observed in the EBAC group after the centralized procurement policy was adjusted, with prices dropping from 206 RMB in stage 1 (1 January 2024–20 September 2025) to 122.25 RMB in stage 2 (21 September 2025–30 November 2025). The cost-effectiveness between the two stages before and after the price adjustment was compared.

The cost-effectiveness ratio (CER) was calculated as the medication cost per percentage point of successful eradication, using the following formula [23]:

CER = Total medication costs/Effectiveness (%).

The incremental cost-effectiveness ratio (ICER) was calculated by dividing the difference in medication costs by the difference in eradication rates between the two groups [23].

ICER = (Cost_VA − Cost_EBAC)/(Effectiveness_VA − Effectiveness_EBAC)

A probabilistic sensitivity analysis (PSA) was conducted by Monte Carlo simulation across 1000 trials to evaluate the influence of uncertainty in the decision tree model. The uncertainty had a beta distribution in clinical probabilities and accuracies and a gamma distribution in costs. A cost-effectiveness acceptability curve (CEAC) and an ICE scatterplot were generated across willingness-to-pay (WTP) thresholds.

### 2.5. Statistical Analysis

Efficacy was evaluated using both intention-to-treat (ITT) and per-protocol (PP) analyses. The ITT group included all enrolled patients; those who discontinued treatment or switched to a different regimen were regarded as treatment failures. The PP group excluded patients who did not complete the full 14-day course or who deviated from the protocol. Continuous variables are described as mean ± standard deviation (SD) and categorical variables as counts (percentages). Categorical variables were compared across groups using the chi-square (χ^2^) test. Both univariate and multivariate logistic regression analyses were performed to identify factors independently associated with eradication failure. Variables with *p* < 0.05 in univariate analysis were entered into the multivariate model, and odds ratios (ORs) with 95% CIs were calculated. Noninferiority was assessed using a one-sided U-test with a 95% CI for the risk difference between groups. Noninferiority was declared if the lower limit of the 95% CI exceeded −10%, and the corresponding *p* value was <0.025. For all other comparisons, two-sided *p* values < 0.05 were considered statistically significant. All statistical analyses were performed using SPSS (version 26.0, IBM Corp., Armonk, NY, USA), NCSS (version 2025, NCSS Corp., Kaysville, UT, USA), TreeAge Pro 2022 (TreeAge Software, LLC, Williamstown, MA, USA), or R software (version 4.5.2, R Foundation for Statistical Computing, Vienna, Austria).

## 3. Results

### 3.1. Baseline Characteristics

Of the 1896 patients treated with either VA or EBAC regimens, 535 were included after applying exclusion criteria: 19 patients were under 18 years old, 10 were over 80 years old, 7 had a history of subtotal gastrectomy, 1301 lacked UBT testing after eradication, and 24 were non-naïve to treatment. Among the 535 eligible patients, 299 received VA, and 236 received EBAC.

**Figure 1:**
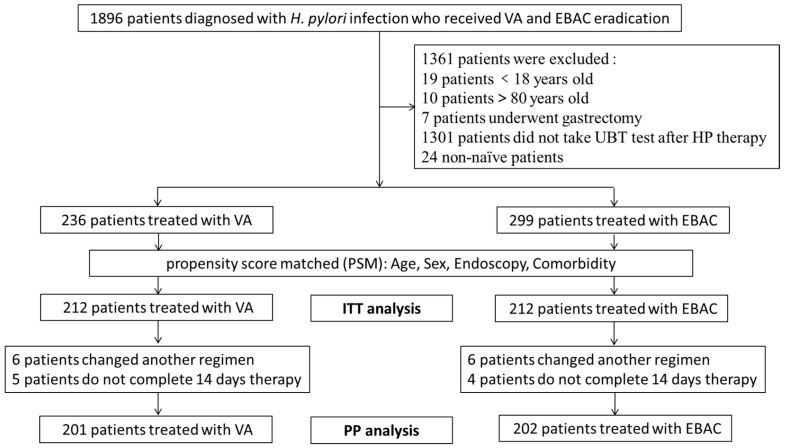
Flowchart of patient enrollment. Abbreviations: *H. pylori*, *Helicobacter pylori*; UBT, urea breath test; VA, vonoprazan–amoxicillin; EBAC, esomeprazole–bismuth–amoxicillin–clarithromycin; ITT, intention to treat; PP, per protocol.

**Figure 2:**
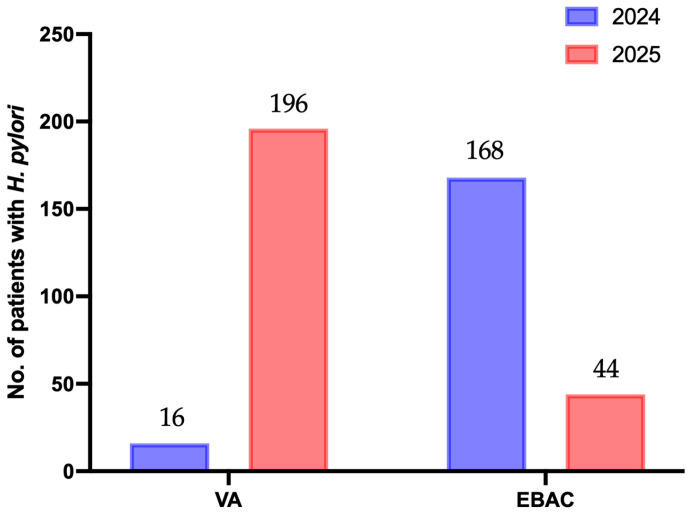
VA versus EBAC for the treatment of *H. pylori* between 2024 and 2025 after PSM. Abbreviations: VA, vonoprazan–amoxicillin; EBAC, esomeprazole–bismuth–amoxicillin–clarithromycin; PSM, propensity score matching.

Baseline characteristics such as age, sex, comorbidity burden, and endoscopic findings (including non-atrophic gastritis, gastric/duodenal ulcers, esophagitis, atrophic gastritis, early gastric cancer, or post-ESD status) were comparable between the VA and EBAC groups, except for the number of comorbidities (*p* = 0.05; Table 2). Following 1:1 PSM adjusted for age, sex, endoscopy, and comorbidities, 424 patients were included in the ITT analysis, with 212 patients assigned to each group. The PP analysis included 201 patients in the VA group and 202 in the EBAC group after excluding those who did not complete the full 14-day regimen or switched therapy (Figure 1). After PSM, the VA and EBAC groups exhibited balanced baseline distributions across all demographic and clinical covariates (*p* > 0.05, Table 2). Since the implementation of the vonoprazan medical insurance reimbursement policy, the number of patients in the VA group who continued to use this drug steadily increased from 16 in 2024 to 196 in 2025. In contrast, the number of patients receiving EBAC decreased from 168 in 2024 to 44 in 2025 (Figure 2).

### 3.2. H. pylori Eradication Rates

The eradication status of *H. pylori* is detailed in Table 3. In the ITT analysis, the *H. pylori* eradication rates were 90.56% (95% CI, 85.88–93.81%) for the VA group and 90.09% (95% CI, 85.33–93.43%) for the EBAC group. In the PP analysis, these rates were 93.03% (95% CI, 88.64–95.80%) and 91.58% (95% CI, 86.93–94.68%), respectively. No significant difference in *H. pylori* eradication rates was observed between the VA and EBAC regimens (*p* > 0.05 in both ITT and PP analyses), with VA demonstrating non-inferiority to EBAC (non-inferiority margin Δ = −10%, *p* < 0.01).

### 3.3. Factors Influencing H. pylori Eradication Failure

Logistic regression analysis was performed to identify risk factors associated with *H. pylori* eradication failure (Table 4). Univariate logistic regression identified that age ≥ 50 years (OR = 2.032; 95% CI: 1.057–3.908; *p* = 0.034), presence of comorbidities (OR = 1.942; 95% CI: 1.009–3.740; *p* = 0.047), and poor compliance (OR = 4.879; 95% CI: 1.746–13.633; *p* = 0.003) were significant predictors of *H. pylori* eradication failure. Multivariate analysis further confirmed that non-compliance remained independently associated with eradication failure (adjusted OR = 5.346; 95% CI: 1.847–15.473; *p* = 0.002).

### 3.4. Cost-Effectiveness Outcomes

The direct medical costs, CERs, and ICERs for the VA regimen compared to EBAC are summarized in Table 5. Cost-effectiveness analysis revealed that the CER for VA therapy exceeded that for EBAC therapy in both periods. In stage 1, the drug cost per patient in the VA group was 96.04 CNY higher than in the EBAC group (302.04 CNY vs. 206 CNY), approximately 1.47 times higher. The ICER for the VA group was −69.59 CNY per 1% increase in eradication rate in the ITT analysis and −640.27 CNY per 1% increase in the PP analysis. In stage 2, the drug cost per patient in the VA group was 179.79 CNY higher than in the EBAC group (302.04 CNY vs. 122.25 CNY), about 2.47 times higher. The ICER for the VA group was 14.95 CNY per 1% increase in eradication rate in the ITT analysis and 13.33 CNY per 1% increase in the PP analysis.

### 3.5. Probabilistic Sensitivity Analysis

**Figure 3:**
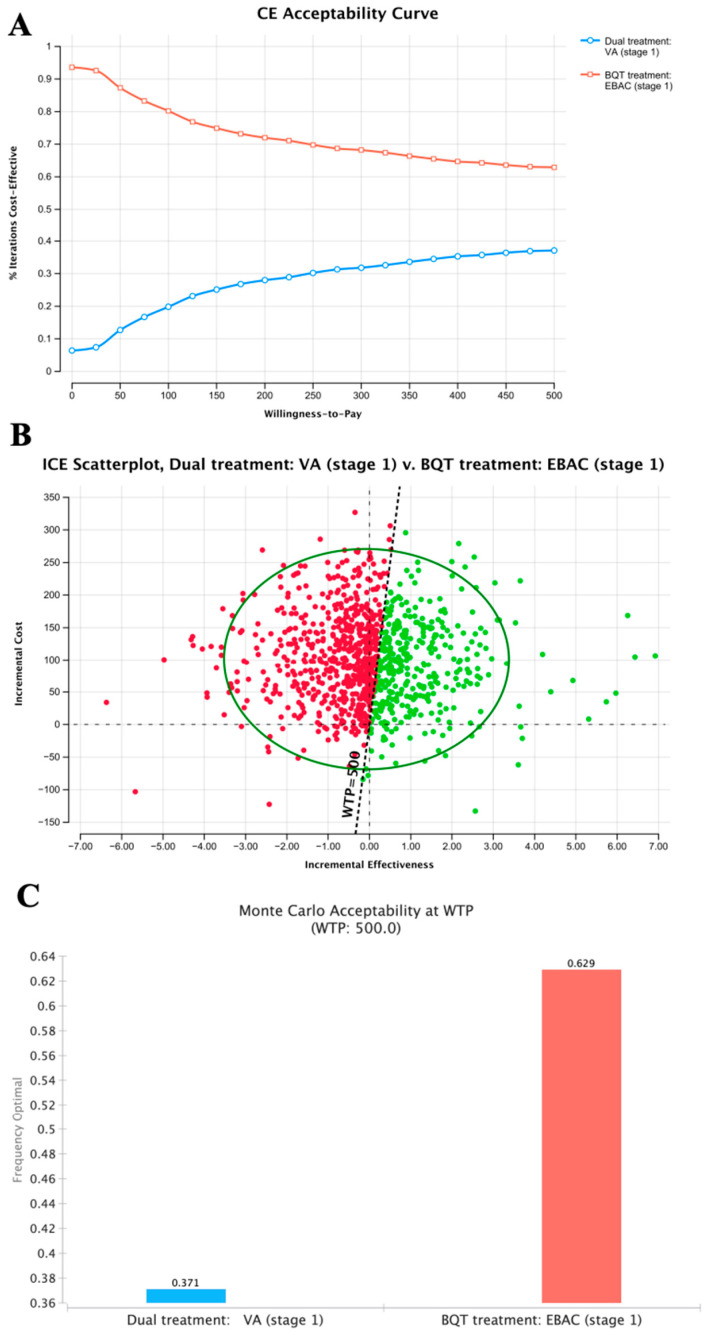
Probabilistic sensitivity analysis of the cost-effectiveness of VA dual therapy versus EBAC bismuth quadruple therapy for *H. pylori* eradication in stage 1: (**A**) Cost-effectiveness acceptability curve (CEAC) showing the probability of each strategy being cost-effective across different willingness-to-pay (WTP) thresholds in stage 1. (**B**) Incremental cost-effectiveness (ICE) scatterplot with 95% CI for *H. pylori*-infected patients in stage. 1. (**C**) Monte Carlo acceptability of the competing strategies for *H. pylori* infection in stage 1. Abbreviations: WTP, willingness to pay; CEAC, cost-effectiveness acceptability curve; ICE, incremental cost-effectiveness; ICER, incremental cost-effectiveness ratio.

The PSA was performed using TreeAge Pro to assess the cost-effectiveness of different *H. pylori* eradication strategies across distinct time periods. The *H. pylori* eradication rate from the PP analysis was used as the baseline for the PSA. The results of the cost-effectiveness analysis of two *H. pylori* eradication strategies during stage 1 are presented in Figure 3. The CEAC showed that the EBAC group consistently possessed higher acceptability across all WTP levels presented. The non-crossing curves confirmed that the EBAC regimen maintained a persistent cost-effectiveness advantage over VA within the 0–500 WTP range (Figure 3A). The ICE scatterplot demonstrated that most points were in the second quadrant, indicating that the VA group had higher costs and lower effectiveness than the EBAC group (Figure 3B). As indicated in Figure 3C, the Monte Carlo acceptability at a WTP threshold of 500 showed that the EBAC group had a higher probability (62.9%) of being the most cost-effective strategy than the VA group (37.1%).

**Figure 4:**
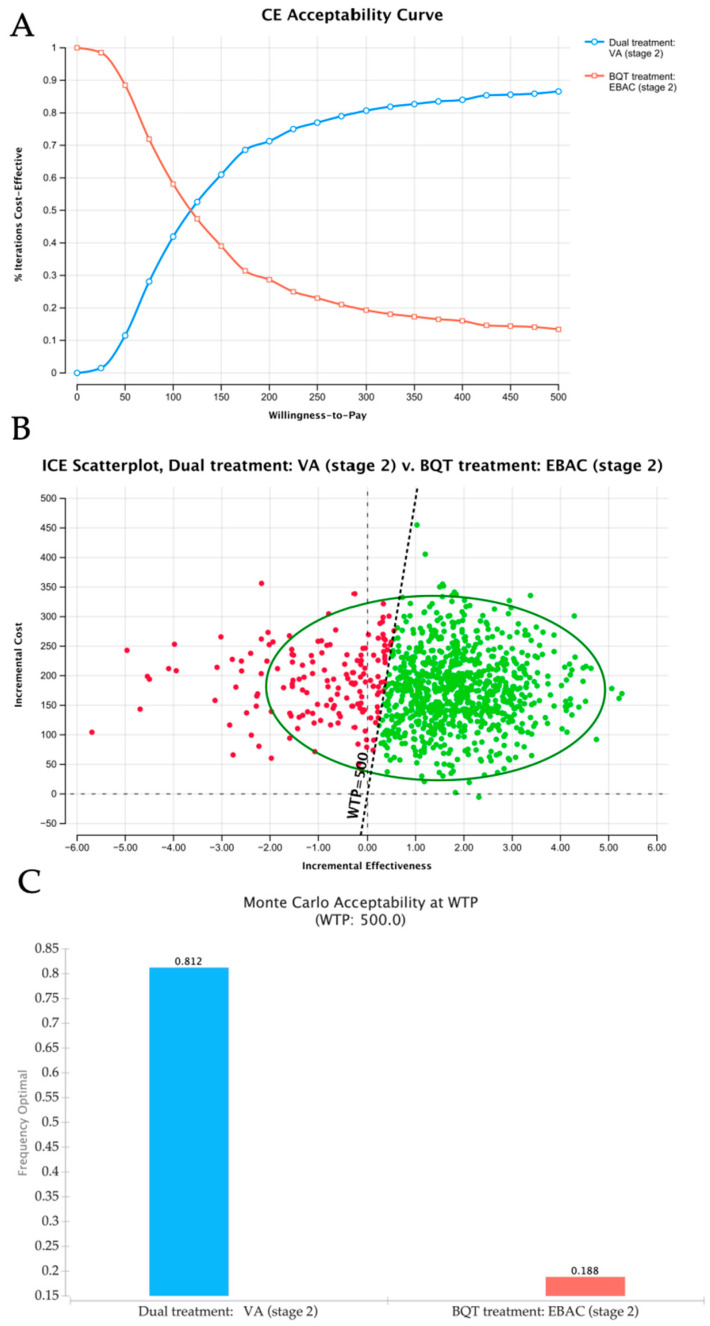
Probabilistic sensitivity analysis of the cost-effectiveness of VA dual therapy versus EBAC bismuth quadruple therapy for *H. pylori* eradication in stage 2: (**A**) CEAC showing the probability of each strategy being cost-effective across different WTP thresholds in stage 2. (**B**) ICE scatterplot with 95% CI for *H. pylori*-infected patients in stage 2. (**C**) Monte Carlo acceptability of the competing strategies for *H. pylori* infection in stage 2. Abbreviations: WTP, willingness to pay; CEAC, cost-effectiveness acceptability curve; ICE, incremental cost-effectiveness; ICER, incremental cost-effectiveness ratio.

The cost-effectiveness outcomes of the two *H. pylori* eradication strategies in stage 2 are illustrated in Figure 4. The CEAC (Figure 4A) indicated that, at WTP thresholds ≤ 125, the EBAC strategy dominated in terms of the cost-effectiveness probability. As the threshold rose, however, the probability of the VA strategy being cost-effective increased, while that of the EBAC strategy decreased. The ICE scatterplot revealed that most simulations for the VA group fell in the first quadrant, indicating that the VA regimen was associated with both higher costs and greater effectiveness than the EBAC group (Figure 4B). As shown in Figure 4C, the Monte Carlo acceptability analysis at a WTP threshold of 500 revealed that the VA group had a substantially higher probability of being cost-effective (81.2%) than the EBAC group (18.8%).

## 4. Discussion

This propensity score-matched retrospective study demonstrates that VA dual therapy achieves favorable efficacy for *H. pylori* eradication comparable to traditional EBAC quadruple therapy, with both regimens achieving >90% in ITT (90.56% vs. 90.09%) and PP (93.03% vs. 91.58%) analyses. There was no statistically significant difference in eradication rates between the high-dose dual therapy with the VA regimen and EBAC therapy. These findings indicate that VA dual therapy and EBAC quadruple therapy are effective for initial eradication.

In alignment with the 2022 Chinese Consensus, antimicrobial susceptibility testing is not recommended as a routine test for patients receiving initial *H. pylori* eradication therapy [11]. Both clinical practice guidelines recommend BQT as first-line treatment, particularly in regions where clarithromycin resistance exceeds 15% [11,17]. According to large-scale local surveillance data from Ningbo (2017 to 2021), the antibiotic resistance rates of *H. pylori* to amoxicillin and clarithromycin were 9.25% and 38.48%, respectively, and the dual-resistance rate to both antibiotics was 1.52% [24]. Our center is in a region with high clarithromycin resistance, and BQT is the first-line regimen for initial *H. pylori* treatment. Our study confirmed that EBAC, as a standard quadruple therapy, achieved an *H. pylori* eradication rate of over 90%, supporting its role as a recommended first-line regimen for *H. pylori* infection. The escalating challenge posed by antibiotic resistance has emerged as the most significant obstacle. In regions with high clarithromycin resistance, BQT maintains an acceptable *H. pylori* eradication rate through several synergistic mechanisms. First, in addition to protecting and repairing the gastric mucosa, bismuth directly disrupts bacterial cell walls and biofilms, disarming resistant strains and reversing their resistance to clarithromycin. *H. pylori* rarely develops resistance to bismuth [25]. Second, when clarithromycin is less effective, amoxicillin remains a first-line agent, offering a very high eradication rate and a low resistance rate of only 3.0% in China [26]. Third, PPIs potently inhibit gastric acid secretion, creating a more favorable intragastric pH environment for all active drugs, which is an important prerequisite for the action of bismuth and the antibiotics [5]. The eradication of *H. pylori* is a synergistic effort that depends on PPIs for adequate acid suppression as the foundational step, antibiotics as the primary therapeutic agents, and bismuth as an adjunct to enhance effectiveness [27]. Clinical studies from China further support the efficacy of this approach. An RCT from Fujian Province reported that a 14-day BQT regimen (esomeprazole 20 mg twice daily + potassium bismuth citrate 240 mg twice daily + amoxicillin 1 g twice daily + clarithromycin 500 mg twice daily) achieved mITT and PP eradication rates of 91.00% and 90.81%, respectively [23]. In Zhejiang Province, which has a high rate of clarithromycin resistance [24,26], a multicenter RCT reported mITT and PP eradication rates of 91.5% and 91.3%, respectively, for a 14-day quadruple regimen (rabeprazole 10 mg, amoxicillin 1 g, clarithromycin 500 mg, and colloidal bismuth 200 mg, all twice daily) [28].

Concurrently, high-dose dual therapy has undergone renewed evaluation; its rationale centers on sustaining intragastric pH > 6 through intensive PPI dosing (e.g., twice-daily high-dose or four-times-daily administration), thereby optimizing the pH-dependent bactericidal activity of amoxicillin [29]. As a new class of acid-suppressing agents, potassium-competitive acid blockers (P-CABs) have gradually replaced traditional PPIs in clinical practice due to their superior acid-suppressing effects [13,19]. The combination of P-CABs with amoxicillin has become an important regimen for *H. pylori* eradication, especially for drug-resistant strains. P-CABs rapidly raise intragastric pH to ≥6 by inhibiting H^+^/K^+^-ATPase [30]. This dual therapy provides rapid onset, sustained acid suppression, and a low risk of resistance, making it a preferred regimen for achieving high *H. pylori* eradication rates, particularly in high-resistance regions and in patients requiring potent acid suppression [31]. VA therapy has garnered attention for its advantages, including satisfactory eradication efficacy, a simplified drug regimen, and a favorable side-effect profile [32]. The VA regimen represents a reliable and effective strategy for *H. pylori* eradication, particularly suitable for patients with suboptimal responses to conventional PPI-based regimens [21]. Our study showed that the VA regimen, as a high-dose dual therapy, also achieved an eradication rate of over 90%. To our knowledge, this study provided novel evidence that VA represents a promising first-line treatment option.

The selection of a first-line eradication regimen for *H. pylori* infection should balance high efficacy, typically defined as an eradication rate of ≥90%, with multiple pragmatic considerations, including drug safety, patient adherence, cost-effectiveness, and proven activity against locally prevalent resistant strains [2]. A Phase III randomized controlled trial in Japan has consistently demonstrated that 14-day vonoprazan-based triple therapy achieves a higher eradication rate than PPI-based triple therapy, thereby providing a foundation for further research into dual therapy [13]. A multicenter, prospective, randomized trial demonstrated that the 14-day VA dual therapy (H-VA-14: vonoprazan 20 mg b.i.d. plus amoxicillin 750 mg q.i.d.) achieved higher eradication rates than the 10-day regimen (H-VA-10) in both ITT (89.5% vs. 86.6%) and PP (94.5% vs. 90.9%) analyses [33]. A meta-analysis of four RCTs with 1807 patients demonstrated that the *H. pylori* eradication rate was significantly higher in the vonoprazan-based high-dose dual therapy group compared to the PPI-based high-dose dual therapy group (88.0% vs. 82.7% by ITT analysis; 92.3% vs. 87.3% by PP analysis) [34]. Consequently, VA dual therapy has consistently demonstrated high eradication rates (>90%) across multiple clinical trials and has been incorporated into national and international guidelines. It is increasingly positioned as a next-generation, simplified first-line option [2,35]. Our ITT analysis showed that the *H. pylori* eradication rates in the VA group were 89.12% and 93.85%, while those in the EBAC group fluctuated significantly, at 90.50% and 81.82%, during stage 1 and stage 2, respectively. The results indicated that the clinical efficacy of the VA group remained relatively stable. However, this did not indicate that the EBAC group was less effective, particularly given the small sample size in stage 2.

*H. pylori* eradication failure is multifactorial, involving antibiotic resistance, adequacy of acid suppression (dependent on PPI/P-CAB choice and dose), bacterial load, and patient-related factors [17,36,37]. Our univariate analysis identified age ≥ 50 years, comorbidities, and patient compliance as potential predictors of *H. pylori* eradication failure. Although advanced age and comorbidities were associated with treatment failure in the univariate analysis, they might not be direct causative factors. For instance, elderly patients frequently experience polypharmacy due to multiple health conditions, which can increase the risk of drug interactions or decreased tolerance to complex treatment regimens, thereby indirectly influencing eradication success [38]. This likely explains why age and comorbidities themselves did not remain in the final model after adjusting for these confounders [36]. A retrospective study reported an OR of 2.165 (95% CI 1.188–3.945, *p* = 0.012) for age > 60 years [39]. However, further multivariate logistic regression analysis revealed that, after controlling for these confounders, only poor compliance remained an independent risk factor (OR 5.346, 95% CI 1.847–15.473, *p* = 0.002).

The medical insurance reimbursement policy has a profound impact on the selection of the therapeutic regimen. Previously, medical insurance reimbursement for vonoprazan was limited to reflux esophagitis. Although previous studies have demonstrated that VA dual therapy achieves *H. pylori* eradication rates comparable to those of bismuth quadruple therapy [28,40], reimbursement restrictions did not cover off-label use for *H. pylori* eradication. As a result, our hospital’s experience with VA dual therapy was extremely limited, with the regimen prescribed almost exclusively to the small number of patients with concurrent reflux esophagitis. A key policy shift on 1 January 2025 made vonoprazan reimbursable for *H. pylori* infection treatment. VA dual therapy subsequently surged in the outpatient department, while traditional quadruple therapy use declined. Concurrently, reforms in centralized drug procurement led to notable price reductions for certain medications, accompanied by fluctuations in eradication rates. Both drug costs and CER of the VA group exceeded those of the EBAC group.

In stage 1, the eradication rate of the VA group was around 90%, but it was still slightly lower than that of the EBAC group. It was at an overall disadvantage, with drug costs 1.47 times higher than those of the EBAC group and corresponding negative ICERs (ITT: −69.59; PP: −640.27). The CEAC and ICE scatterplots from the probabilistic sensitivity analysis confirmed that VA was more costly and less effective. In stage 2, the drug costs of the EBAC group decreased significantly (206 to 122.25 RMB) due to centralized procurement, and the eradication rate also declined to 81.82%, which was much lower than in stage 1. This difference might be due to the small sample size and may not be generalizable, warranting further investigation. However, the VA therapy maintained a stable therapeutic effect. The PSA revealed that the VA group was more costly but also more effective. The EBAC therapy was a particularly cost-effective option for patients with limited financial resources or medical insurance. Meanwhile, the VA regimen offered notable benefits, including reliable therapeutic results, better patient adherence, and fewer side effects [28,41]. As a result, the VA regimen provides sustained and stable therapeutic effects, supported by medical insurance coverage.

This study has several limitations. First, as a single-center retrospective study, it is inevitably affected by recall bias and missing records. The lack of ^13^C/^14^C UBT results for many patients may have introduced selection bias and impacted the accuracy of the reported eradication rate. The main reasons for loss to follow-up included poor patient compliance, inconvenience in returning to the hospital for testing, and limited awareness of the importance of post-eradication evaluation. We used PSM analysis to minimize the potential impact of unmeasured factors on the results. Second, we cannot definitively confirm whether those lost to follow-up had clinical outcomes similar to the included patients; if non-adherent or treatment-failure patients were disproportionately lost, the observed high eradication rate might be overestimated. Third, antibiotic susceptibility testing was not performed during the initial treatment of patients with *H. pylori* infection. We cannot conduct subgroup analyses based on actual resistance status, which may affect the precise interpretation of the causes of treatment failure. Fourth, the study considered only direct drug costs and did not account for indirect costs, such as patient transportation expenses, or long-term costs related to *H. pylori* recurrence or complications. This could result in an incomplete evaluation of the overall economic burden of the two regimens. Fifth, the observation period of this study was relatively short (from January 2024 to November 2025), especially for stage 2, which might limit the external validity of the results. These limitations should be considered when assessing the generalizability and robustness of our findings.

## 5. Conclusions

In conclusion, VA dual therapy is a safe, effective, and patient-friendly first-line treatment for *H. pylori* eradication, with success rates around 90%, similar to traditional BQT. Its superior tolerability and simplified regimen improve adherence, a critical determinant of treatment success. While EBAC therapy remains a highly cost-effective foundational option, especially for patients with financial constraints, the VA regimen provides a compelling alternative amid the growing threat of antimicrobial resistance. The key to successful eradication ultimately rests on a balanced, individualized approach: selecting appropriate antibiotics; achieving effective, sustained acid suppression; and ensuring patient adherence throughout the entire course of therapy.

## Figures and Tables

**Table 1:** Inclusion and exclusion criteria.

Inclusion Criteria	ages 18 to 80 years, male or female
	diagnosis of *H. pylori* infection by ^13^C/^14^C-urea breath test (UBT) or histological staining of biopsy samples
	treatment-naïve
Exclusion Criteria	history of allergy to amoxicillin or other medications
	presence of severe diseases or clinical conditions, such as liver disease, lung disease, kidney disease, metabolic disease, cardiovascular disease, or malignant tumor
	pregnancy or breastfeeding
	previous gastrectomy
	presence of serious gastrointestinal diseases, such as malignant tumors, gastrointestinal bleeding, or Zollinger–Ellison syndrome

**Table 2:** Baseline characteristics of patients before and after propensity score matching.

Variable	Before PSM					After PSM				
	Total (n = 535)	EBAC (n = 299)	VA (n = 236)	Statistic	*p*	Total (n = 424)	EBAC (n = 212)	VA (n = 212)	Statistic	*p*
Age, Mean ± SD	45.89 ± 15.21	45.15 ± 13.91	46.82 ± 16.71	t = −1.233	0.218	45.11 ± 15.32	45.43 ± 14.61	44.80 ± 16.03	t = 0.424	0.671
Sex, n (%)				χ^2^ = 0.323	0.570				χ^2^ = 0.237	0.626
Male	242 (45.23)	132 (44.15)	110 (46.61)			195 (45.99)	95 (44.81)	100 (47.17)		
Female	293 (54.77)	167 (55.85)	126 (53.39)			229 (54.01)	117 (55.19)	112 (52.83)		
Number of Comorbidities, n (%)				χ^2^ = 7.798	0.050				χ^2^ = 1.524	0.677
0	365 (68.22)	209 (69.90)	156 (66.10)			298 (70.28)	152 (71.70)	146 (68.87)		
1	114 (21.31)	68 (22.74)	46 (19.49)			80 (18.87)	39 (18.40)	41 (19.34)		
2	32 (5.98)	14 (4.68)	18 (7.63)			27 (6.37)	14 (6.60)	13 (6.13)		
≥3	24 (4.49)	8 (2.68)	16 (6.78)			19 (4.48)	7 (3.30)	12 (5.66)		
Endoscopy, n (%)				χ^2^ = 11.861	0.105				-	0.998
No endoscopy	210 (39.25)	120 (40.13)	90 (38.14)			174 (41.04)	88 (41.51)	86 (40.57)		
Non-atrophic gastritis	170 (31.78)	106 (35.45)	64 (27.12)			126 (29.72)	62 (29.25)	64 (30.19)		
Gastric ulcer	17 (3.18)	10 (3.34)	7 (2.97)			14 (3.3)	7 (3.30)	7 (3.30)		
Duodenal ulcer and duodenitis	18 (3.36)	11 (3.68)	7 (2.97)			11 (2.59)	5 (2.36)	6 (2.83)		
Concomitant gastric and duodenal ulcers	12 (2.24)	6 (2.01)	6 (2.54)			10 (2.36)	6 (2.83)	4 (1.89)		
esophagitis	9 (1.68)	5 (1.67)	4 (1.69)			8 (1.89)	4 (1.89)	4 (1.89)		
atrophic gastritis	94 (17.57)	39 (13.04)	55 (23.31)			76 (17.92)	38 (17.92)	38 (17.92)		
Early gastric cancer or ESD	5 (0.93)	2 (0.67)	3 (1.27)			5 (1.18)	2 (0.94)	3 (1.42)		

Abbreviations: PSM, propensity score matching; ESD, endoscopic submucosal dissection.

**Table 3:** Eradication rates of the two groups.

Analysis	VA	EBAC	Difference (95% CI)	* *p* for Difference	^†^ *p* for Non-Inferiority
ITT	90.56% (192/212)	90.09% (191/212)	0.47% (−5.28% to 6.23%)	0.869	0.003
95% CI	85.88–93.81%	85.33–93.43%			
PP	93.03% (187/201)	91.58% (185/202)	1.45% (−3.92% to 6.86%)	0.585	0.0001
95% CI	88.64–95.80%	86.93–94.68%			

Abbreviations: CI, confidence interval. * *p* for difference; ^†^ *p* for non-inferiority.

**Table 4:** Univariate and multivariable analyses of risk factors for *H. pylori* eradication failure.

Variables	No.	Failure, No. (%)	Univariate Analysis		Multivariate Analysis	
			OR (95%CI)	*p*	OR (95%CI)	*p*
Age						
<50	243	17 (7.0)	1.0		1.0	
≥50	181	24 (13.3)	2.032 (1.057–3.908)	0.034	1.590 (0.773–3.267)	0.207
Sex						
Male	195	17 (8.7)	1.0			
Female	229	24 (10.5)	1.226 (0.638–2.355)	0.541		
Comorbidities						
No	296	23 (7.8)	1.0		1.0	
Yes	128	18 (14.1)	1.942 (1.009–3.740)	0.047	1.801 (0.865–3.750)	0.116
Endoscopy						
No endoscopy	179	8 (4.6)	1.0			
Non-atrophic gastritis	126	16 (12.7)	5.187 (0.518–51.912)	0.161		
Peptic ulcer	35	3 (8.6)	1.719 (0.181–16.359)	0.638		
Esophagitis	8	2 (25.0)	2.667 (0.221–32.178)	0.440		
Atrophic gastritis	76	11 (14.5)	0.750 (0.050–11.211)	0.835		
Early gastric cancer or ESD	5	1 (20.0)	1.477 (0.151–14.480)	0.738		
Symptoms						
No	254	19 (7.5)	1.0			
Yes	170	22 (12.9)	1.839 (0.962–3.513)	0.065		
Treatment regimen						
EBAC	212	21 (9.9)	1.0			
VA	212	20 (9.4)	0.869 (0.497–1.805)	0.869		
Compliance						
No	19	6 (31.6)	4.879 (1.746–13.633)	0.003	5.346 (1.847–15.473)	0.002
Yes	405	35 (8.6)	1.0		1.0	

Abbreviations: OR, odds ratio.

**Table 5:** Cost-effectiveness of different periods in ITT and PP analyses.

Period	Analysis	Group	Total Cost (CNY)	Eradication Rates (%)	CER	ICER
Stage 1	ITT	VA	302.04	89.12% (131/147)	3.39	−69.59
		EBAC	206	90.50% (181/200)	2.28	
	PP	VA	302.04	91.97% (126/137)	3.28	−640.27
		EBAC	206	92.12% (175/190)	2.24	
Stage 2	ITT	VA	302.04	93.85% (61/65)	3.22	14.95
		EBAC	122.25	81.82% (9/11)	1.49	
	PP	VA	302.04	95.31% (61/64)	3.27	13.33
		EBAC	122.25	81.82% (9/11)	1.49	

Stage 1: From 1 January 2024 to 20 September 2025; stage 2: From 21 September 2025 to 30 November 2025. Abbreviations: CER, cost-effectiveness ratio; ICER, incremental cost-effectiveness ratio.

## Data Availability

No new data were created or analyzed in this study. Data sharing is not applicable to this article.

## Abbreviations

The following abbreviations are used in this manuscript:

VA	vonoprazan–amoxicillin
BQT	bismuth quadruple therapy
PPI	proton pump inhibitor
ITT	intention to treat
PP	per protocol
HDDT	high-dose dual therapy
P-CAB	potassium-competitive acid blocker
UBT	urea breath test
PSM	propensity score matching
CER	cost-effectiveness ratio
ICER	incremental cost-effectiveness ratio
CEAC	cost-effectiveness acceptability curve
ICE	incremental cost-effectiveness
WTP	willingness to pay
